# Consensus on the standard terminology used in the nutrition care of adult patients with chronic kidney disease

**DOI:** 10.1590/2175-8239-JBN-2020-0210

**Published:** 2021-04-09

**Authors:** Cristina Martins, Simone L. Saeki, Marcelo Mazza do Nascimento, Fernando M. Lucas, Ana Maria Vavruk, Christiane L. Meireles, Sandra Justino, Denise Mafra, Estela Iraci Rabito, Maria Eliana Madalozzo Schieferdecker, Letícia Fuganti Campos, Denise P. J. van Aanholt, Ana Adélia Hordonho, Marcia Samia Pinheiro Fidelix

**Affiliations:** 1 Associação Brasileira de Nutrição, Curitiba, PR, Brasil.; 2 Sociedade Brasileira de Nefrologia, Comitê de Nutrição, Curitiba, PR, Brasil.; 3 Sociedade Brasileira de Nutrição Parenteral e Enteral, Curitiba, PR, Brasil.; 4 Consórcio de Pesquisa e Implementação da TPCN no Brasil, Curitiba, PR, Brasil.; 5 Grupo de Trabalho Internacional da NCPT, Subcomitê Internacional da Academy of Nutrition and Dietetics (Academy) para a TPCN, Curitiba, PR, Brasil.; 6 Instituto Cristina Martins de Educação e Pesquisa em Saúde, Curitiba, PR, Brasil.; 7 Universidade Federal do Paraná, Curitiba, PR, Brasil.; 8 Hospital das Clínicas da Universidade Federal de Minas Gerais/Grupo Nefroclínicas, Belo Horizonte, MG, Brasil.; 9 Hospital e Maternidade Municipal de São José dos Pinhais, São José dos Pinhais, PR, Brasil.; 10 University of Texas Health Science Center, School of Nursing, San Antonio, USA.; 11 Universidade Federal do Paraná, Complexo do Hospital de Clínicas da UFPR, Curitiba, PR, Brasil.; 12 Universidade Federal Fluminense, Rio de Janeiro, RJ, Brasil.; 13 Federación Latinoamericana de Terapia Nutricional, Nutrición Clínica y Metabolismo, Ecuador.; 14 Universidade Estadual de Ciências da Saúde, Hospital Escola Hélvio Auto e Hospital Metropolitano de Alagoas, Maceió, AL, Brasil.; 15 Centro Universitário CESMAC, Maceió, AL, Brasil.

**Keywords:** Nutritional Sciences, Malnutrition, Renal Insufficiency, Chronic, Food Assistance, Terminology, Ciências da Nutrição, Desnutrição, Insuficiência Renal Crônica, Assistência Alimentar, Terminologia

## Abstract

This nutrition consensus document is the first to coordinate the efforts of three professional organizations - the Brazilian Association of Nutrition (Asbran), the Brazilian Society of Nephrology (SBN), and the Brazilian Society of Parenteral and Enteral Nutrition (Braspen/SBNPE) - to select terminology and international standardized tools used in nutrition care. Its purpose is to improve the training delivered to nutritionists working with adult patients with chronic kidney disease (CKD). Eleven questions were developed concerning patient screening, care, and nutrition outcome management. The recommendations set out in this document were developed based on international guidelines and papers published in electronic databases such as PubMed, EMBASE(tm), CINHAL, Web of Science, and Cochrane. From a list of internationally standardized terms, twenty nutritionists selected the ones they deemed relevant in clinical practice involving outpatients with CKD. The content validity index (CVI) was calculated with 80% agreement in the answers. The Grading of Recommendations, Assessment, Development and Evaluation (GRADE) framework was used to assess the strength of evidence and recommendations. A total of 107 terms related to Nutrition Assessment and Reassessment, 28 to Diagnosis, nine to Intervention, and 94 to Monitoring and Evaluation were selected. The list of selected terms and identified tools will be used in the development of training programs and the implementation of standardized nutrition terminology for nutritionists working with patients with chronic kidney disease in Brazil.

## Resumo

Este consenso representa a primeira colaboração entre três organizações profissionais com foco em nutrição: Associação Brasileira de Nutrição (Asbran), Sociedade Brasileira de Nefrologia (SBN) e Sociedade Brasileira de Nutrição Parenteral e Enteral (Braspen/SBNPE), com o objetivo de identificar a terminologia e instrumentos padronizados internacionalmente para o processo de cuidado em nutrição. O foco é facilitar a condução de treinamentos de nutricionistas que trabalham com pacientes adultos com doenças renais crônicas (DRC). Foram levantadas onze questões relacionadas à triagem, ao processo de cuidado e à gestão de resultados em nutrição. As recomendações foram baseadas em diretrizes internacionais e em bancos de dados eletrônicos, como PubMed, EMBASE(tm), CINHAL, Web of Science e Cochrane. A partir do envio de listas de termos padronizados internacionalmente, vinte nutricionistas especialistas selecionaram aqueles que consideraram muito claros e relevantes para a prática clínica com pacientes ambulatoriais com DRC. Foi calculado o Índice de Validade de Conteúdo (IVC), com 80% de concordância nas respostas. O Grading of Recommendations, Assessment, Development and Evaluation (GRADE) foi usado para atribuir força de evidência às recomendações. Foram selecionados 107 termos de Avaliação e Reavaliação, 28 de Diagnóstico, 9 de Intervenção e 94 de Monitoramento e Aferição em Nutrição. A lista de termos selecionados e identificação de instrumentos auxiliará no planejamento de treinamentos e na implementação de terminologia padronizada em nutrição no Brasil, para nutricionistas que trabalham com pacientes renais crônicos.

## Introduction

Nutritional status plays a fundamental role in the health and clinical outcomes of individuals with chronic kidney disease (CKD). Malnutrition is a highly prevalent condition closely linked to adverse clinical outcomes and increased hospitalization, complication, and death rates in this population^1^^,^ 2. The pathogenesis of malnutrition in CKD is multifactorial and complex, and its main causes revolve around reduced food intake, nutrient anabolism, and hypercatabolism^1^^-^ 3.

The use of standardized terminology and tools is required to clearly document the impact of nutrition care and capture the specificity of prescribed care measures. Standardization enhances search capabilities in electronic databases and communication of medical facts in electronic patient charts. Examples of standardization in nephrology include the KDIGO (Kidney Disease Improving Global Outcomes) and the KDOQI (Kidney Disease Outcomes Quality Initiative). A recent publication on nomenclature for kidney disease attempted to improve communication between health care workers and the population^4^.

Using predetermined terms and accurate data enables the comprehension of the links connecting problems, specific interventions, and significant outcomes reached in nutrition and health. Standardized terminology and tools provide for a consistent means to capture care actions and describe positive outcomes from nutritional and health care interventions.

The International Statistical Classification of Diseases and Related Health Problems 10^th^ Revision (ICD-10) of the World Health Organization is the official system used to designate diagnostic and medical procedure codes. Although some concepts from nutrition have been incorporated in the ICD-10, they are insufficient when it comes to characterizing nutritional problems and specific interventions prescribed by nutritionists. The work of nurses and physicians encompasses different areas, and the needs of both are different from the needs of other health care workers.

Some international nomenclature systems used in electronic patient charts offer some potential to include nutrition terminology. One of them is the SNOMED-CT (Systematized Nomenclature of Medicine-Clinical Terms) (http://www.snomed.org/), maintained by the International Health Terminology Standards Development Organization (IHTSDO) since 2007. The system was initially developed to encompass diseases, but eventually progressed considerably to include terms from other areas of knowledge, such as nursing and nutrition. The SNOMED-CT is considered the most complete and accurate terminology database. Brazil joined SNOMED International in 2018. Therefore, the standardization of clinical terminology - including nutrition care - has become a matter of national interest.

In Nephrology, patients often move between outpatient and inpatient care. Therefore, the standardization of nutrition terms and tools, particularly considering the use of electronic charts and records, optimizes the sharing of data and the communication between institutions, improves data quality and intervention outcomes, increases patient safety by allowing seamless care, decreases rework, and saves time and money. However, nutrition terminology has not been standardized in Brazil and electronic patient charts have not been developed to allow the entry of structured data (without free text). These relevant processes are challenging, and require good planning and strong solutions.

The purpose of this consensus document was to identify selected terms in nutrition from international nomenclature to improve the training of nutritionists working with patients with kidney disease in Brazil. It also aimed to find validated screening and malnutrition diagnostic tools that might be incorporated in the practice of this group of nutritionists. Therefore, the target audience of this consensus document is nutritionists working with adult individuals (age > 18 years) with CKD in outpatient care with non-dialysis dependent kidney disease, on hemodialysis (HD), on peritoneal dialysis (PD), and kidney transplant patients.

## Questions

Eleven questions covering three topics were defined, as described below. The 2019 edition of the Nutrition Care Process Terminology (NCPT) translated into Portuguese after validation by two reviewers, both nutritionists who have Portuguese as their mother tongue, in line with the criteria set out by the Academy of Nutrition and Dietetics (Academy), was used as a reference.

**Topic: Screening and referral systems for patients with CKD**
1. Which malnutrition screening tool should be used?

**Topic: Nutrition care process for patients with CKD**
2. Should the nutrition care process (NCP) and the Nutrition Care Process Terminology (NCPT) be standardized?
3. Which Nutrition Assessment and Reassessment standardized terms are deemed very relevant by Brazilian nutritionists?
4. Which Nutrition Diagnosis standardized terms are deemed very relevant by specialist nutritionists?
5. Should malnutrition be defined based on etiology?
6. Which malnutrition diagnostic tool should be used?
7. Which Nutrition Intervention standardized terms are deemed very relevant by specialist nutritionists?
8. Which reference standards for daily nutrient and food intake are recommended?
9. Which Nutrition Monitoring and Evaluation standardized terms are deemed very relevant by specialist nutritionists?

**Topic: Outcome management system for patients with CKD**
10. Which format should be used in the documentation of NCP data?
11. Which indicators should be used in nutrition outcome management?

Experienced nutritionists (with at least two years of practice with outpatients with CKD) were selected to answer the questions on the selection of terms. Specialist nutritionists were emailed the list of terms of the NCPT and were asked to individually select terms they deemed very relevant in CKD outpatient clinical practice. The answers were collected in a spreadsheet containing all NCPT codes.

The content validity index (CVI) was calculated to determine and quantify content validity^5^^,^ 6. The CVI comprises a scale from 1 to 4, in which 1 - not relevant; 2 - somewhat relevant; 3 - quite relevant; 4 - highly relevant. On account of the great number of standardized terms, specialist nutritionists were asked to pick only the terms rated as "4" in the CVI scale (number of answers rated as "4" / total number of answers).

Since more than six specialists answered the questionnaire, an agreement rate of 80% was stipulated as the threshold to characterize answers representing the group's opinions^7^^,^ 8.

### Levels of evidence

The recommendations made in this document were derived and adapted from consensus documents and international guidelines cited in the References section. Whenever questions could not be answered with the aid of international guidelines or consensus documents, searches were made (by August 31, 2020) in electronic databases - PubMed, EMBASE(tm), CINHAL, Web of Science, and Cochrane - for relevant papers. Evidence cited in guidelines, consensus documents, and literature were discussed and listed in a table of levels of evidence, with recommendations produced subsequently. Agreement within the working group was used as a reference in cases in which evidence was inconclusive or insufficient.

The Grading of Recommendations, Assessment, Development and Evaluation (GRADE)^9^ framework was used to assess the strength of evidence (Chart 1). The GRADE framework has been extensively used and is deemed a methodologically sound, easy-to-use tool.

**Chart 1:** Grading of Recommendations, Assessment, Development and Evaluation (GRADE)^9^ Framework

Level of evidence	Definition of evidence	Notes	Information source
A-High	There is confidence that the true effect is close to the estimated effect	It is unlikely that additional papers might modify the confidence in the estimation of the effect.	Well-designed clinical trials with significant populations.
			In some cases, well-designed observational studies with consistent findings.*
B-Moderate	There is moderate confidence in the estimated effect	Future papers might modify the confidence in the estimation of the effect and modify the estimation itself.	Clinical trials with mild** limitations.
			Well-designed observational studies with consistent findings.*
C-Low	Confidence in the estimated effect is limited	Future papers will probably have a significant impact on the confidence of the estimation of effect.	EClinical trials with moderate** limitations
			Comparative observational studies: cohort and case control studies.
D-Very Low	Confidence in the estimation of effect is very limited. There is an important degree of uncertainty in the findings.	Any estimation of effect is limited..	Clinical trials with severe limitations.**
			Comparative observational studies with limitations.**
			Observational studies without comparisons.***
			Expert opinions.

https://www.gradeworkinggroup.org/

* Cohort studies without methodological limitations, with consistent findings presenting large effect size and/or dose-response gradients.

** Limitations: study design biases, surrogate endpoints, or compromised external validity.

*** Case series and case reports.

Strength of recommendation (Chart 2) was assessed based on discussions including expert opinions, cost-effectiveness of the recommendations, costs and reviewed supporting evidence, followed by the use of the Delphi method and voting, until agreement was reached.

**Chart 2:** Strength of recommendation

Strength of recommendation	
1-Strong	We recommend/do not recommend it
2-Weak	We suggest/do not suggest it

## Recommendations For The Screening And Referral System

**Table:** 

Recommendation 1
The Malnutrition Screening Tool (MST) should be used to screen patients with CKD at risk of malnutrition. Screening should be performed at least monthly.
Level of Evidence A, Strength 1

### Comments

The MST supports the NCP. Screening helps to identify patients at risk of malnutrition and may be performed in any clinical practice environment. In addition to nutritionists, trained individuals (physicians, nurses, nutrition technicians, interns, family members, patients, etc.) may conduct screening sessions^10^^,^ 11. Screening may be useful for patients assessed, diagnosed, and treated by a nutritionist. Screened patients may be prescribed nutrition care.

Numerous screening tools have been developed and/or validated for patients with CKD. They include the Geriatric Nutritional Risk Index (GNRI), validated for patients on HD^12^ and PD^13^; the Nutritional Risk Screening 2002 (NRS-2002) validated for patients on HD^14^; and the Renal Nutrition Screening Tool (R-NST), validated for hospitalized individuals with kidney disease^15^.

Ideally, a tool should function regardless of underlying disease, age, or site of application to acknowledge risk of malnutrition. In other words, it should not address specific patient populations, but allow for universal use instead. Therefore, this consensus supports the systematic review published by Skipper et al^16^. and the more recent position of the Academy^10^, which have described the MST (Chart 3) as the tool with the best validity, agreement, and reliability, regardless of age, medical history, or site where the patient is offered care. The MST has been validated and shown good generalization for patients with acute disease, on long-term treatment, rehabilitation, outpatients, and individuals treated for cancer in at least nine different countries^17^^-^ 35.

**Chart 3:** Malnutrition Screening Tool - MST

Questions	Score
1) Have you recently lost weight without trying?	
• No	0
• Unsure	2
2) If yes, how much weight have you lost (kg)?	
• 1-5	1
• 6-10	2
• 11-15	3
• > 15	4
• Unsure	2
3) Have you been eating poorly because of a decreased appetite?	
• No	0
• Yes	1
Interpretation: ≥ 2 = risk of malnutrition	Total Score: __________

Adapted from Fergunson et al., 1999^38^.

The KDOQI does not indicate a specific tool to screen patients for risk of malnutrition, although it states screening for malnutrition should be performed at least twice a year for patients with CKD stages 3-5, individuals on dialysis, and patients in post-kidney transplant care^36^.

The simplicity of the MST allows the tool to be put to use by patients themselves, their family members and caretakers, in addition to health care workers. A study revealed that the MST is a reliable, valid tool that accurately identifies risk of malnutrition when used by individuals with cancer in outpatient care compared to a situation in which nutritionists use the tool to identify patients at risk^37^.

Since malnutrition is a significant risk for patients with CKD strongly related to morbidity and mortality, we recommend that screening be performed at least once a month. Patients can self-administer the screening tool or have their caretakers involved. This consensus group also suggests that campaigns should be organized to build the awareness of patients and health care workers over the need to administer the MST frequently.

## Recommendations for the nutrition care process

**Table:** 

Recommendation 2
The Nutrition Care Process (NCP) and the Nutrition Care Process Terminology (NCPT) should be standardized for patients with CKD.
Level of evidence B, Strength 1

### Comments

The nutrition care process (NCP) adopted by the Academy^39^ is a systematic, complete, thorough approach to collect, verify, categorize, interpret, and document data. It comprises four steps, each organized based on categories, classes, and subclasses^40^. The steps are Nutrition Assessment and Reassessment; Diagnosis; Intervention; and Monitoring and Evaluation. Nutritionists are required to go through the four steps of the NCP. Each step must be completed before moving on to the next.

**Figure 1:**
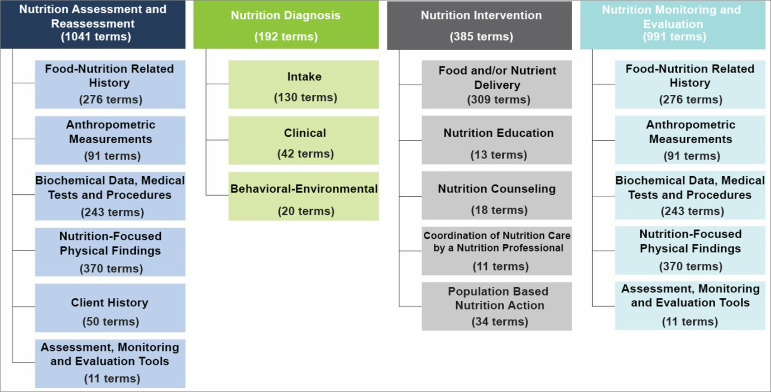
Standardized categories of the four steps of the Nutrition Care Process version 2019,40 with the number of terms for each step.

Nutrition Care Process Terminology (NCPT) is the professional language used to standardize and encode specific terms^40^. It is the controlled glossary that supplements the NCP. The NCPT is a system hierarchically organized (Figure 1) to produce accurate, specific descriptions of the services delivered by nutritionists. The NCPT aims to improve the quality of care and related outcomes^40^.

Use of the NCPT has been reported in instructional practices and environments^41^^-^ 47 in different parts of the world^48^^,^ 49. Implementation has been linked to numerous improvements. The NCPT helps to develop a common framework for routine care and research in nutrition. Standardized terminology may also encourage critical thinking and more focused and productive data documentation, potentially improving communication between health care workers.

The Academy, alongside other international organizations, has made significant efforts to establish the NCPT as a global language. Terms are updated once a year and made available on a web platform. The NCPT has also been adjusted to meet the requirements of international health systems and evidence-based guidelines^46^^,^ 50 ^-^ 52. Since 2011, the terms of the NCP steps have been included in interdisciplinary international standards such as the SNOMED-CT^53^. They reflect the standardized clinical terminology used in electronic patient chart systems used in several countries. Although they have been translated into several languages and dialects, a study showed that the NCP and the NCPT have not been fully adopted in the clinical practice of nutritionists working with patients with CKD, mostly due to lack of information^54^.

In 2014, the ASBRAN took the first steps toward international standardization and published the Guidelines for Systematization of Nutrition Care (Manual Orientativo: Sistematização do Cuidado em Nutrição - SICNUT)^55^. The SICNUT contains the diagnostic nutrition recommendations proposed by the Academy. The ASBRAN entered into a partnership with the Academy in 2015, and has a seat in the International NCPT Subcommittee. In 2016-2018, the NCP and NCPT manuals were translated into Portuguese and validated as per the criteria set out by the Academy. In 2020, the Brazilian Consortium for Research and Implementation of the NCPT was created, with the Federal University of Paraná (UFPR) as the first Reference Center for research and training on the NCPT in the nation. The development of consensus documents within specialties in nutrition is one of the elements in the strategic plan developed by the Consortium.

The standardization of the NCPT in Brazil will also help to implement the Academy of Nutrition and Dietetics Health Informatics Infrastructure (ANDHII®), a web-based data acquisition platform.53 The ANDHII® is based on the NCPT, and can be easily integrated into other healthcare information systems at a relatively low cost. It has been used in education, research, medical practices, and public health centers in the United States and various other countries^53^. Using one single information system will undoubtedly lead to significant savings of time and resources in dialysis clinics, hospitals, outpatient clinics, medical practices, and other healthcare services. It may also encourage additional local and global research in nutrition and health.

**Table:** 

Recommendation 3
From a total of 1,041 internationally standardized terms in Nutrition Assessment and Reassessment, 107 should be included in the initial training program for nutritionists working with patients with CKD in Brazil.
Level of evidence C, Strength 1

### Comments

Assessment and Reassessment involves a systematic approach to collecting, categorizing, and summarizing nutritional data. The goal is to describe the nutritional status and the problems related to nutrition and their etiology^40^. Findings are compared to criteria or standards, reference frameworks (national, international or regulatory), or health care provider and patient-defined goals. Collected data may also be used to manage the quality of nutrition care.

Etiology guides the intervention plan designed to improve patient nutrition status. The search for etiology is an important element in Nutrition Assessment and Reassessment, since it is particularly useful in connecting diagnosis and intervention^53^. The NCPT standardizes and encodes etiology, thus allowing the identification of the types of intervention that might address specific problems. Each diagnosis in nutrition may stem from different etiologies.

The NCPT encompasses a large number of terms that support the work of nutritionists in every area in which their presence is needed, including neonatology, public health, sports, and medical practices. Since it has not been widely used in a number of areas, including nephrology, starting from a shorter list of terms may facilitate professional training and the implementation of the NCPT. Table 1 presents a selection of terms in Assessment and Reassessment deemed essential by nutritionists specialized in working with patients with CKD.

**Table:** 

Recommendation 4
From a total of 1,041 internationally standardized terms in Nutrition Assessment and Reassessment, 107 should be included in the initial training program for nutritionists working with patients with CKD in Brazil.
Level of evidence C, Strength

**Table 1:** Nutrition Assessment and Reassessment Terms deemed essential by nutritionists specialized in kidney disease

CATEGORIES/TERMS	CODE	CATEGORIES/TERMS	CODE
DOMAIN: FOOD-NUTRITION RELATED HISTORY (FH)		ANTHROPOMETRIC MEASURES (AD)	
Total energy intake	FH-1.1.1.1	Measured height	AD-1.1.1.1
Oral fluids	FH-1.2.1.1	Knee height	AD-1.1.1.10
Amount of food	FH-1.2.2.1	Measured body weight	AD-1.1.2.1
Types of food/meals	FH-1.2.2.2	Reported usual body weight	AD-1.1.2.5
Formula/enteral nutrition solution	FH-1.3.1.1	Estimated dry weight	AD-1.1.2.10
Oral fat intake	FH-1.5.1.1	Pre-dialysis body weight	AD-1.1.2.15
Total protein intake	FH-1.5.3.1	Post dialysis body weight	AD-1.1.2.16
High biological value protein intake	FH-1.5.3.2	Weight gain	AD-1.1.4.1
Total fiber intake	FH-1.5.6.1	Weight loss	AD-1.1.4.2
24-h potassium intake	FH-1.6.2.2.5	Percent weight change	AD-1.1.4.3
24-h phosphorus intake	FH-1.6.2.2.6	Measured interdialytic weight gain	AD-1.1.4.4
Modified diet prescription	FH-2.1.1.2	Body mass index	AD-1.1.5.1
Food allergies	FH-2.1.2.5	Percent body fat	AD-1.1.7.1
Food intolerance	FH-2.1.2.6	Mid-arm muscle circumference	AD-1.1.7.9
Food preferences	FH-4.3.12	Tricipital skinfold thickness	AD-1.1.7.11
Physical ability to feed independently	FH-7.2.2	Arm circumference	AD-1.1.7.19
BIOCHEMICAL DATA, MEDICAL TESTS AND PROCEDURES (BD)		Obstipation	PD-1.1.5.9
Creatinine	BD-1.2.2	Reduced appetite	PD-1.1.5.10
Glomerular filtration rate	BD-1.2.4	Diarrhea	PD-1.1.5.11
Sodium	BD-1.2.5	Early satiety	PD-1.1.5.12
Potassium	BD-1.2.7	Epigastric pain	PD-1.1.5.13
Serum calcium	BD-1.2.9	Heartburn	PD-1.1.5.18
Phosphorus	BD-1.2.11	Liquid stool	PD-1.1.5.22
Parathyroid hormone	BD-1.2.13	Nausea	PD-1.1.5.24
Fasting glucose	BD-1.5.1	Vomiting	PD-1.1.5.27
HbA1c	BD-1.5.3	Pitting edema +1	PD-1.1.6.1
C-reactive protein	BD-1.6.1	Pitting edema +2	PD-1.1.6.2
Serum cholesterol	BD-1.7.1	Pitting edema +3	PD-1.1.6.3
HDL cholesterol	BD-1.7.2	Pitting edema +4	PD-1.1.6.4
LDL cholesterol	BD-1.7.3	Anasarca	PD-1.1.6.5
Serum triglycerides	BD-1.7.7	Ankle edema	PD-1.1.6.6
Hemoglobin	BD-1.10.1	Amputated foot	PD-1.1.7.1
Hematocrit	BD-1.10.2	Amputated hand	PD-1.1.7.2
Serum ferritin	BD-1.10.10	Amputated leg	PD-1.1.7.3
Serum iron	BD-1.10.11	Anuria	PD-1.1.9.2
Total iron-binding capacity	BD-1.10.12	Alopecia	PD-1.1.10.2
Transferrin saturation	BD-1.10.13	Ageusia (loss of taste)	PD-1.1.13.1
Albumin	BD-1.11.1	Angular cheilitis	PD-1.1.13.2
Urine output	BD-1.12.4	Muscle atrophy	PD-1.1.14.1
Urine microalbumin	BD-1.12.10	Muscle cramps	PD-1.1.14.3
24-h urine protein	BD-1.12.12	Dizziness	PD-1.1.16.12
NUTRITION-FOCUSED PHYSICAL FINDINGS (PD)		Dry skin	PD-1.1.17.8
Asthenia	PD-1.1.1.1	Skin pruritus	PD-1.1.17.38
Obesity	PD-1.1.1.10	Toothlessness	PD-1.1.18.10
Excess subcutaneous fat	PD-1.1.2.2	Dysphagia	PD-1.1.19.3
Subcutaneous fat loss	PD-1.1.2.3	Swallowing disorders	PD-1.1.19.10
Central adiposity	PD-1.1.2.4	Blood pressure	PD-1.1.21.1
Abdominal distension	PD-1.1.5.3		
CLIENT HISTORY* (CH)		Immune (ex.: food allergies)	CH-2.1.8
Age	CH-1.1.1	Medical treatment/therapy	CH-2.2.1
Gender	CH-1.1.2	Surgical therapy	CH-2.2.2
Sex	CH-1.1.3	End-of-life palliative care	CH-2.2.3
Mobility	CH-1.1.12	Socioeconomic factors	CH-3.1.1
Client main nutrition complaint	CH-2.1.1	Social and medical support	CH-3.1.4
Cardiovascular	CH-2.1.2	ASSESSMENT, MONITORING, AND EVALUATION TOOLS (AT)	
Gastrointestinal	CH-2.1.5	Subjective Global Assessment (SGA) score	AT-1.1

* Note: Client, in standardized terminology, refers to individuals, groups, populations, and support structures and individuals.

### Comments

Nutrition diagnosis states a specific problem that may be resolved or improved by means of intervention by a nutritionist.

The adoption of diagnostic language is a central element in documentation, since it standardizes the terminology used to name patient health problems and needs^56^. Studies are currently in progress to validate the contents of the section on diagnosis in the NCPT. An early study has tested the content for validity^57^. Validation has also been performed by nutritionists specialized in pediatrics^58^, gerontology^59^, and oncology^60^. Although additional refinement is needed, the terminology has been considered acceptable. Table 2 presents the terms selected by expert nutritionists.

**Table:** 

Recommendation 5
The definition of protein-energy malnutrition may be standardized for patients with CKD based on etiology and association with inflammation, as follows: 1) associated with chronic disease or condition with ongoing inflammation; 2) associated with chronic disease with minimal or undetected inflammation; 3) associated with acute disease or injury with severe inflammation; and 4) associated with chronic low food intake unrelated to the disease.
Level of evidence B, Strength 1

**Table 2:** Nutrition Diagnosis Terms deemed essential by nutritionists specialized in chronic kidney disease

CATEGORIES/TERMS	CODE	CATEGORIES/TERMS	CODE
INTAKE		CLINICAL - NC	
Increased energy expenditure	NI-1.1	Biting/chewing impairment	NC-1.2
Sub-optimal energy intake	NI-1.2	Altered gastrointestinal function	NC-1.4
Excessive energy intake	NI-1.3	Altered nutrition-related workup results (specify)	NC-2.2
Sub-optimal oral intake	NI-2.1	Low weight	NC-3.1
Excessive fluid intake	NI-3.2	Non-volitional weight loss	NC-3.2
Sub-optimal protein-energy intake	NI-5.2	Overweight/obesity	NC-3.3
Excessive fat intake	NI-5.5.2	Malnutrition (undernutrition)	NC-4.1
Sub-optimal protein intake	NI-5.6.1	Malnutrition related to chronic disease or condition	NC-4.1.2
Excessive protein intake	NI-5.6.2	Moderate malnutrition related to chronic disease or condition	NC-4.1.2.1
Excessive carbohydrate intake	NI-5.8.2	Severe malnutrition related to chronic disease or condition	NC-4.1.2.2
Sub-optimal fiber intake	NI-5.8.5	Moderate malnutrition related to acute disease or injury	NC-4.1.3.1
Sub-optimal mineral intake (specify)	NI-5.10.1	Severe malnutrition related to acute disease or injury	NC-4.1.3.2
Excessive mineral intake (specify)	NI-5.10.2	BEHAVIORAL - ENVIRONMENTAL - NB	
Potassium	NI-5.10.2.5	Physical inactivity	NB-2.1
Phosphorus	NI-5.10.2.6		

### Comments

In nephrology, there is a vast number of terms for malnutrition, including uremic malnutrition^61^, kidney cachexia/uremic cachexia^62^, sarcopenia in kidney disease^63^^,^ 64, malnutrition, inflammation, and atherosclerosis (MIA) syndrome^65^^-^ 68 or malnutrition-inflammation complex syndrome (MICS), protein-energy malnutrition^69^ and protein-energy wasting^70^^,^ 71. Each definition of malnutrition validated for this group of patients includes different sets of criteria. Therefore, prevalence may vary and comparison is potentially hampered. Besides, with standardization in mind, the definition of malnutrition cannot apply only to individuals with CKD. In order to strengthen medical practice and research, validated terms and criteria applicable beyond kidney disease must be defined.

PEW and sarcopenia are the terms more commonly related to malnutrition in individuals with CKD. In the NCPT, sarcopenia is not a diagnosis of malnutrition, but rather an element related to signs and symptoms gathered during Assessment and Reassessment. PEW has not been included in the NCPT, and since it applies only to patients with CKD, it cannot be included in SNOMED.

The NCPT separates the diagnosis of malnutrition into three categories based on etiology, as established in the international standardization proposal put forward by the Academy/ASPEN (American Society of Parenteral and Enteral Nutrition) in 2012^72^. Focus in etiology is given to the inflammatory process, a common finding in CKD closely related to malnutrition and patient death.

In 2017, the guidelines of the ESPEN (European Society for Clinical Nutrition and Metabolism) posited that malnutrition might be further divided into four categories:^73^ 1) associated with chronic disease or condition with ongoing inflammation; 2) associated with chronic disease with minimal or undetected inflammation; 3) associated with acute disease or injury with severe inflammation; and 4) associated with chronic low food intake unrelated to the disease. The definitions and categories in the ESPEN apply to patients with CKD in various stages of the disease and care center types (e.g.: clinics, hospitals, outpatient clinics). Therefore, they may be recommended in standardization.

**Table:** 

Recommendation 6
The Subjective Global Assessment (SGA) is the best validated protein-energy malnutrition diagnostic tool for patients with CKD. The Malnutrition Clinical Characteristics (MCC) is an objective tool validated for different patient populations that may also be used with individuals with CKD.
Level of evidence A for the SGA and B for the MCC; Strength 1

### Comments

Several malnutrition diagnostic tools have been proposed and validated for patients with CKD. The SGA has been validated multiple times for all stages of CKD^74^^,^ 75.

In addition, a number of tools stemmed from the traditional SGA, including the Patient Generated Subjective Global Assessment (PG-SGA), validated for individuals on HD^76^, and some added specific data, such as the 7-point SGA^77^^,^ 78. This scale disregards edema and considers years on dialysis and presence of comorbidities instead. Another offshoot is the Malnutrition-Inflammation Score (MIS)^78^^-^ 83, in which three items were added: the body mass index (BMI), serum albumin, and total iron-binding capacity.

Additionally, results from the Mini Nutritional Assessment Long-Form (MNA-LF)^84^^,^ 85 and the Nutritional Competence Score (NCS)^86^^,^ 87 have been associated with mortality of patients with CKD. Associations have been reported between the Objective Score of Nutrition on Dialysis (OSND) and the MIS^88^. Significant correlations have been described between the Integrative Clinical Nutrition Dialysis Score (ICNDS) and the SGA^8^^)(^ 9. PEW criteria have also been used to diagnose malnutrition^90^. Associations have been reported between PEW and SGA results and mortality of patients on dialysis^91^.

The KDOQI recommends the 7-point SGA for patients with CKD stage 5 and the MIS for individuals on HD and patients in post-transplant care^36^. However, since they are specific for individuals with CKD, these tools cannot meet the universality requirement. The NCPT recommends the SGA, the PG-SGA, and the MNA-LF for adult populations. The ESPEN^73^ recommends these tools for patient populations. However, if standardization is the target, using different tools becomes unpractical.

Although adjustments are often made to existing tools and new ones are constantly being developed, the traditional SGA is cited in every guideline, since it has been validated for different populations and care center types, even after modifications. The lack of universal acceptance of the SGA might be due to uncertainties tied to its subjective nature.

The Global Leadership Initiative on Malnutrition (GLIM)^92^ involved the four largest international clinical nutrition societies and developed a consensus document on practical indicators to diagnose various forms of malnutrition in different target populations and care center types. In the GLIM, at least one phenotypic criterion and one etiologic criterion must be met for an individual to be diagnosed with malnutrition. Phenotypic criteria include non-volitional weight loss, low body mass index, and reduced muscle mass. Etiologic criteria include reduced food intake or assimilation and inflammation or disease burden. The GLIM was not designed as a measurement tool, but as a diagnostic framework. However, its criteria and severity cutoff points have not been validated^93^. With the exception of kidney transplant patients and individuals with early-stage CKD, the inclusion of BMI cutoff points may decrease specificity. Evidence indicates the existence of an epidemiologically counter-intuitive association (a negative association) between having a high BMI and mortality of patients with kidney disease and individuals on HD in particular^36^, which might hamper the creation of different BMI cutoff points for different patient populations. Therefore, the BMI cannot be regarded as a universal criterion.

The MCC is a less subjective tool than the SGA^72^. It uses the three categories of malnutrition based on etiology (Chart 4). The MCC does not include the BMI or serum albumin as indicators, but agrees with the GLIM criteria and is based on a consistent definition of malnutrition. Besides, all indicators included in the MCC were recommended by the KDOQI^36^ for the assessment of malnutrition of patients with CKD.

**Chart 4:** Clinical characteristics of malnutrition in adults: Academy and ASPEN criteria

Clinical indicators	Malnutrition related to acute disease or injury				Malnutrition related to chronic disease or condition				Malnutrition related to social/environmental circumstances			
	Moderate malnutrition		Severe malnutrition		Moderate malnutrition		Severe malnutrition		Moderate malnutrition		Severe malnutrition	
1. Decreased energy intake	< 75% of the estimated energy requirement for > 7 days.		≤ 50% of the estimated energy requirement for ≥ 5 days.		< 75% of the estimated energy requirement for ≥ 1 month.		< 75% of the estimated energy requirement for ≥ 1 month		< 75% of the estimated energy requirement for ≥ 3 months		≤ 50% of the estimated energy requirement for ≥ 1 month	
2. Perda de peso	%	Time	%	Tempo	%	Tempo	%	Tempo	%	Tempo	%	Tempo
	1-2	1 week	>1-2	1 week	5	1 month	>5	1 month	5	1 month	>5	1 month
	5	1 month	>5	1 month	7,5	3 months	>7,5	3 months	7,5	3 months	>7,5	3 months
	7,5	3 months	>7,5	3 months	10	6 months	>10	6 months	10	6 months	>10	6 months
					20	1 year	>20	1 year	20	1 year	>20	1 year
3. Body Fat Loss	Mild		Moderate		Mild		Severe		Mild		Severe	
4. Muscle Mass Loss	Mild		Moderate		Mild		Severe		Mild		Severe	
5. Fluid Retention	Mild		Moderate to Severe		Mild		Severe		Mild		Severe	
6. Hand Grip Strength	-		Decreased		-		Decreased		-		Decreased	

* Note: At least two indicators or clinical characteristics must be present for an individual to be diagnosed with malnutrition. Adapted from the Consensus statement: Academy of Nutrition and Dietetics and American Society for Parenteral and Enteral Nutrition: characteristics recommended for the identification and documentation of adult malnutrition (undernutrition), 2012^72^.

Studies reported satisfactory levels of accuracy and moderate agreement for the MCC compared to the SGA in adult hospitalized patients^94^, individuals with severe conditions in general, trauma^95^, and surgery patients^96^. In regard to outcomes, the MCC predicted longer hospitalization times^97^ and higher care costs^98^. In patients submitted to abdominal cancer surgery, higher degrees of malnutrition assessed by the MCC were associated with longer hospitalization, higher cost of care, higher hospital mortality, more severe complications, and higher readmission rates^99^. Similar results were obtained in retrospective studies with inpatients in general^100^^,^ 101. Malnutrition assessed by the MCC was also associated with long term mortality (within up to two years) of elderly patients with pneumonia^102^. Studies performed in ICU settings showed that MCC results were good predictors of death and length of hospitalization^103^^,^ 104. A prospective study enrolling 600 adult and elderly hospitalized subjects reported concurrent and predictive validity for the MCC even without using the hand grip strength test^105^. The causes of hospitalization revolved primarily around chronic ailments including cancer, heart and lung diseases, and gastrointestinal disorders. The MCC showed good agreement and satisfactory levels of accuracy compared to the SGA for endpoints length of hospitalization, hospital deaths, readmission, and mortality within six months of discharge. In elderly patients on follow-up care after acute disease, MCC results were also associated with length of hospitalization and functional capacity^106^.

To our knowledge, no studies have been published on the applicability of the MCC to patients with CKD. However, after analyzing the literature, it is likely that this tool might be valid for the population at hand.

**Table:** 

Recommendation 7
From a total of 385 internationally standardized terms in Nutrition Intervention, nine should be included in the initial training program for nutritionists working with patients with CKD in Brazil.
Level of evidence C, Strength 1

### Comments

Table 3 lists the terms used in Nutrition Intervention the experts selected. Nutrition Intervention in the NCP includes a set of behaviors and specific action either performed, delegated, coordinated, or recommended by a nutritionist^53^. Intervention helps patients to resolve or improve from their problem. It is subdivided into two interconnected stages: planning and implementation.

**Table 3:** Nutrition Intervention Terms deemed essential by nutritionists specialized in chronic kidney disease

CATEGORIES/TERMS	CODE	CATEGORIES/TERMS	CODE
FOOD AND/OR NUTRIENT SUPPLY (ND)		Diet with fluid restriction	ND-1.2.8.2
Increased energy diet	ND-1.2.2.1	Low potassium diet	ND-1.2.11.5.2
Increased protein diet	ND-1.2.3.2	Low phosphorus diet	ND-1.2.11.6.2
Low carbohydrate diet	ND-1.2.4.3	Low sodium diet	ND-1.2.11.7.2
Low simple carbohydrate diet	ND-1.2.4.3.2	Change in enteral nutritional prescription	ND-2.1.1

The planning stage includes the dietary prescription and the nutrition intervention goals. It is preferable that nutritionists and their patients define the two jointly. Goals must be attainable, measurable, and related to the condition the patient has been diagnosed with. The development of the care plan must be based on evidence-based care guidelines and other references, so that the expected patient-focused results are achieved in each point of the nutrition diagnosis. The plan also sets out the time and frequency of care, along with the resources needed to achieve the established goals.

During implementation, the nutritionist in charge defines the interventions, selects appropriate strategies, discusses ideas with the patient, and implements the plan. Based on the patient's condition, length of treatment and monitoring are defined and additional materials are developed.

Several intervention strategies may be recommended for patients with CKD, with the primary goal of preventing or reversing situations of malnutrition. Individualized ongoing education and counseling on nutrition are of the essence to prevent malnutrition and fluid, vitamin, and mineral imbalances in patients with CKD^107^.

**Table:** 

Recommendation 8
The KDOQI Nutrition guidelines should be used as the standard reference for daily nutrient intake for patients with CKD. Tools My Plate, Mediterranean Diet Pyramid, and the DASH Diet may be recommended as references for food choices and may be adjusted to patients in various stages of CKD. Individual goals must be established based on professional judgment.
Level of evidence B, Strength 1

### Comments

The standard reference for daily nutrient intake guides Assessment and Reassessment (quantitative adjustment analysis) and Intervention (diet planning and prescription) in the NCP. Nutritionists may select the most adequate standard reference to define individualized goals based on professional judgment.

For healthy individuals and conditions lacking specific nutrient intake recommendations, the most widely used standard reference is the DRIs (Dietary Reference Intakes)^108^^-^ 114. For metabolically stable patients with CKD, this consensus document recommends the Clinical Practice Guideline for Nutrition in Chronic Disease^36^ (Chart 5) as the standard reference for daily nutrient intake. The guidelines are part of the KDOQI developed by the National Kidney Foundation and the Academy.

**Chart 5:** References for daily nutrient intake for patients with chronic kidney disease

Energy and Nutrients	Non-dialytic	Hemodialysis	Peritoneal Dialysis
Energy (kcal/kg of current or ideal weight in case of obesity of very low weight)	25-35	25-35	25-35 (diet + dialysate)
Protein (kcal/kg of current or ideal weight in case of obesity of very low weight)	0.55-0.60 with mixed diet or 0.28-0.43 with vegetarian diet + 0.28-0.43 with essential amino acid or keto acid supplementation Patients with diabetes: 0.6-0.8	1.0-1.2	1.0-1.2
Sodium (mg)	< 2,300	< 2,300	< 2,300
Potassium (mg)	Adjusted to maintain normal serum levels	Adjusted to maintain normal serum levels	Adjusted to maintain normal serum levels
Fluids (mL)^a^	Usually without restrictions	Adjusted for interdialytic weight gain (ideal: 2.5-4%)	Usually without restrictions
Phosphorus (mg)	Adjusted to maintain normal serum levels	Adjusted to maintain normal serum levels	Adjusted to maintain normal serum levels
Calcium (mg)	If patient is not taking vitamin D: 800-1,000 (including diet, supplements, and calcium-based binders)	Adjusted (diet, supplements, and calcium-based binders) considering the use of vitamin D to maintain normal serum levels	Adjusted to maintain normal serum levels

Adapted from KDOQI, 2020;^36^

a Opinion.

Tools such as My Plate, the Mediterranean Diet Pyramid^115^, and the DASH (Dietary Approaches to Stop Hypertension) Diet may be used as references for daily food intake for patients with CKD stages 1-5. The same tools may be easily adjusted to meet the needs of patients on HD or PD.

**Table:** 

Recommendation 9
From a total of 991 internationally standardized terms in Nutrition Monitoring and Evaluation, 94 should be included in the initial training program for nutritionists working with patients with CKD in Brazil
Level of evidence C, Strength 1

### Comments

Monitoring and Evaluation is the last step in the NCP^53^. It includes three elements: monitoring, measurement, and evaluation of the changes in signs and symptoms (Assessment and Reassessment indicators).

Nutrition Monitoring and Evaluation includes the examination of post-intervention outcomes, the selection of quality indicators derived from evidence-based, best practice guidelines^53^. Indicators use available data to provide quantitative measures of the desired targets. The need for Reassessment is defined during Monitoring and Evaluation^53^.

The standardized terminology for Nutrition Monitoring and Evaluation is the same used in Assessment and Reassessment (Table 1), with the exception of the terms used in Client History (50 terms).

## Recommendations for the outcome management system

**Table:** 

Recommendation 10
The acronym ADIME (Assessment, Diagnosis, Intervention, and Monitoring/Evaluation) should be used as a reference to document the Nutrition Care Process of patients with CKD.
Level of evidence C, Strength 1

### Comments

The NCP requires documentation so that patient care can be monitored and assessed and proper support given to outcome management systems. Documents in standardized format optimize quality management and enable performance assessment.

The elements comprised in the ADIME acronym are as follows: "Assessment/ Reassessment (A), Diagnosis (D), Intervention (I), and Monitoring/Evaluation (ME)"^39^. In "D", it is recommended that a PES (problem; etiology; signs and symptoms) statement be produced^39^. The term "related to" should be placed next to the problem label to identify the cause of the problem. The etiology (cause) is made up of the factors that contribute to the existence of the problem.

The identification of the etiology leads to the selection of intervention, which purpose is to resolve the nutrition problem. Signs and symptoms (indicators) are the elements that define whether the patient presents with a specific nutrition problem. They are connected to etiology by the words "as evidenced by."

The ADIME acronym has not been officially standardized to document he NCP, but it has been recommended on account of its practicality and ease-of-use. Regardless of format, documentation must be clear, accurate, concise, specific, limited to one problem at a time, and precisely related to etiology and information collected during nutrition assessment. It should contain as little free text as possible to facilitate comparisons and analysis of performance indicators.

**Table:** 

Recommendation 11
Outcome management in malnutrition must split patients into age ranges. Other indicators directly related to nutrition interventions are interdialytic weight gain, phosphorus, calcium, 25-hydroxyvitamin D, potassium, serum bicarbonate and glucose, or glycosylated hemoglobin.
Level of evidence A, Strength 1

### Comments

The Outcome Management System also supports the NCP^53^ and is operated by individuals with different backgrounds. It is responsible for supporting ongoing quality improvement and is extremely important in any care environment.

An Outcome Management System defines the indicators used to reflect the current status of a problem to compare it against a predefined ideal status or established realistic improvement goal. Goals must be identified based on the reality of each institution. They must be challenging, but possible to achieve. They must also be constantly adjusted (reviewed) against achieved results.

Calculations and comparison of management indicators identify the actions required to improve the quality of the services delivered. Key and specific indicators in nutrition must reflect solely what must be improved through the work of nutritionists. Other indicators must be considered jointly with a multidisciplinary team for opportunities to improve service in general.

The standardization of the NCPT in electronic patient chart systems allows documentation in a structured format. Workflows and tools used in this task have been published for adult and pediatric practices^116^. Data entries with minimal free text (structured patient chart) allows for quick access, less ambiguity and more specificity, and confinement within evidence-based parameters. Consequently, outcome management is facilitated, care efficiency increased, and nutrition outcomes are improved^45^.

The Outcome Management System monitors the success of the implementation of the NCP and provides input and advice. The goal is to optimize the delivery of care by focusing on process quality, effectiveness, and efficiency. Management tools enable compliance verification and the identification of nonconformities. Chart 6 includes items usually available in practices involving patients with CKD and closely related to the NCP. Most have had their relevance acknowledged in the KDOQI^36^ and were included in the recommendations of the guidelines of the American Diabetes Association^117^. Since nutrition is a high risk factor in this population, it is recommended that it be analyzed in terms of severity for different age ranges.

**Chart 6:** Quality management indicators recommended for nutrition care of patients with chronic kidney disease

Indicators	Percent Adequacy
Severe malnutrition	% Adequacy = number of patients aged 50 years or less with severe malnutrition x 100/total number of patients
Severe malnutrition	% Adequacy = number of patients aged 50-80 years with severe malnutrition x 100/total number of patients
Severe malnutrition	% Adequacy = number of patients aged 80+ years with severe malnutrition x 100/total number of patients
Mild/moderate malnutrition	% Adequacy = number of patients aged 50 years or less with mild/moderate malnutrition x 100/total number of patients
Mild/moderate malnutrition	% Adequacy = number of patients aged 50-80 years with mild/moderate malnutrition x 100/total number of patients
Mild/moderate malnutrition	% Adequacy = number of patients aged 80+ years with mild/moderate malnutrition x 100/total number of patients
Interdialytic weight gain	% Adequacy = number of patients with IWG between 2.5% and 4.0% x 100/total number of patients
Serum phosphate	% Adequacy = number of patients with serum phosphate between 3.5 and 5.5 mg/dL x 100/total number of patients
Serum calcium	% Adequacy = number of patients with calcium between 8.4 and 9.5 mg/dL x 100/total number of patients
Serum 25(OH)D	% Adequacy = number of patients with 25(OH)D ≥ 30 ng/mL x 100/total number of patients
Serum potassium	% Adequacy = number of patients with serum potassium between 3.5 e 5.5 mg/dL x 100/total number of patients
Serum bicarbonate	% Adequacy = number of patients with serum bicarbonate between 24 and 26 mmol/L x 100/total number of patients
Fasting glucose or glycosylated hemoglobin (HbA1c)	% Adequacy = number of patients with serum glucose between 70 and 99 mg/dL x 100/total number of patients
	% Adequacy = number of patients with HbA1c between 6.5% and 7.0% x 100/total number of patients

Adapted from: KDOQI, 2020^36^, American Diabetes Association, 2019^117^ and Opinion.

## Conclusion

Standardizing terminology does not mean that the same care measures will be provided to every patient. Tailoring care to patient needs and values and using the best evidence available to make decisions are still required.

However, standardization inevitably introduces changes to practice. It is a relevant factor in clinical assessment and facilitates the documentation and management of the outcomes derived from nutrition care. Standardization allows the introduction of information systems in data collection and analysis, thereby strengthening the bridges between technology, practice, and research.

Once the learning curve has been overcome, the implementation of the NCPT and screening and assessment tools introduces significant opportunities to improve the effectiveness of nutrition services. Care is improved in terms of service and outcomes; communication between health care workers and institutions is enhanced; priorities in intervention plans are optimally assigned; realistic, measurable goals can be set; the documentation of patient charts is improved; services are better managed and outcomes more clearly understood; payments for procedures is facilitated; specific contributions coming from nutritionists in patient care are viewed more clearly and appreciated by the care team and the community.

To sum up with, standardization in nutrition brings significant progress in practice, education, research, and regulation. It is certainly the most effective way to show the impact nutrition care has in the health of individuals with CKD.
